# Experience, Outcomes, and Insights on Endoscopic Colonic Polyp Excision: A Retrospective Cohort Study

**DOI:** 10.7759/cureus.75979

**Published:** 2024-12-18

**Authors:** Mohamed Hassan, Amr K Ebrahim, Ahmed A Saad, Ahmed Moustafa, Mahmud Riad, Dinesh Balasubramaniam

**Affiliations:** 1 General Surgery, Maidstone and Tunbridge Wells NHS Trust, Maidstone, GBR; 2 Surgery, Maidstone and Tunbridge Wells Hospital, Maidstone, GBR; 3 General Surgery, Cairo University, Cairo, EGY; 4 Surgery, Maidstone and Tunbridge Wells Hospitals, Maidstone, GBR; 5 Colorectal Surgery, Maidstone and Tunbridge Wells Hospitals, Maidstone, GBR

**Keywords:** cold snare polypectomy, colon polypectomy, colonscopy, colorectal cancer, conventional endoscopic mucosal resection (cemr), hot snare polypectomy

## Abstract

Introduction

Colorectal cancer (CRC) continues to pose a major public health challenge, ranking among the most common malignancies globally and being a leading cause of cancer-related mortality. Most CRCs originate from adenomatous polyps, underscoring the importance of detecting and removing these precancerous growths as a key preventive measure against CRC. In particular, large colonic polyps (≥10 mm) warrant special attention due to their increased risk of progressing to malignancy compared to smaller polyps.

This retrospective study aims to evaluate the outcomes of large polyp excision within a single NHS trust. By analysing patient demographics, polyp characteristics, procedural details, and clinical outcomes, this study seeks to provide comprehensive data on the efficacy and safety of endoscopic polyp excision.

Patients and methods

This retrospective cohort study was conducted at Maidstone and Tunbridge Wells NHS Trust, a district general hospital in Tunbridge Wells, England, with data collected for all patients who had endoscopic polypectomy from 2010 to 2022. Data collected from medical records included age, sex, polyp size and characteristics, and previous polyp removal attempts.

Results

A total of 350 participants were included in this study. The cohort comprised 224 males (64%) and 126 females (36%), with a mean age of 70 ± 12 years. Complications were observed in eight participants (2.3%), while 340 participants (97.7%) did not experience any complications. Participants who experienced complications had a mean polyp size of 49.38 ± 7.3 mm, whereas those without complications had a mean polyp size of 44.85 ± 16.5 mm (p = 0.440). All complications occurred in participants with polyps ≥40 mm. However, this association was not statistically significant (p = 0.352).

Conclusion

Our study has shown that large polyps can be safely excised in a local district hospital. The incidence of complications is related to the polyp size. A cutoff size of 40 mm was found to be related to increased complications. However, this was still statistically insignificant and won’t affect the efficacy and safety of these procedures being carried out in a local district hospital, decreasing the load on tertiary and specialised hospitals.

## Introduction

Colorectal cancer (CRC) remains a significant public health challenge, ranking among the most prevalent malignancies globally and being a leading cause of cancer-related mortality [1]. Most CRCs originate from adenomatous polyps, highlighting the importance of detecting and removing these precancerous growths as a primary preventive measure against CRC [2]. Early detection of polyps and timely intervention through colonoscopy have been pivotal in reducing CRC-related mortality rates [3]. In particular, large colonic polyps (≥10 mm) warrant special attention owing to their increased risk of progressing to malignancy compared with smaller polyps [4].

Endoscopic polypectomy has become the standard procedure for the removal of colonic polyps, significantly reducing the incidence and mortality associated with CRC [5]. However, the excision of large polyps presents unique challenges. These polyps often exhibit characteristics such as extensive size, difficult location, and sessile or flat morphology, complicating removal and increasing the risk of procedural complications such as bleeding and perforation [6]. Historically, large polyps were often referred for surgical resection, but advancements in endoscopic techniques have enabled endoscopists to manage these lesions more effectively [7].

Techniques such as endoscopic mucosal resection (EMR) and endoscopic submucosal dissection (ESD) have been developed to manage large and complex colonic polyps. EMR involves injecting a solution to lift the polyp from the submucosa, allowing for its snare resection, while ESD allows for en-bloc resection by dissecting the submucosal layer beneath the lesion [8]. Although these methods are technically demanding, they have shown promising results regarding complete resection rates and safety profiles [9,10].

This retrospective study aims to evaluate the outcomes of large polyp excision within a single NHS trust. By analysing patient demographics, polyp characteristics, procedural details, and clinical outcomes, the study seeks to provide comprehensive data on the efficacy and safety of endoscopic polyp excision.

## Materials and methods

This retrospective cohort study was conducted at Maidstone and Tunbridge Wells NHS Trust, a district general hospital in Maidstone, England, with data collected for all patients who had an endoscopic polypectomy from 2010 to 2022. Data collected from medical records included age, sex, polyp size and characteristics, and previous polyp removal attempts.

Endoscopic mucosal resection 

Endoscopic mucosal resection was performed for all the patients involved in this study using the following technique.

Lesion Identification and Marking

Target polyps were identified using high-definition white-light endoscopy, with narrow-band imaging (NBI) employed for enhanced visualisation when required. In cases of poorly visible margins, submucosal injection was preceded by circumferential marking using a cautery device.

Submucosal Injection

A solution comprising saline, diluted epinephrine (1:100,000), and a small amount of methylene blue dye was injected beneath the lesion to lift the polyp and facilitate safe resection while minimising the risk of perforation.

Snare Resection

A polypectomy snare was positioned around the elevated lesion and closed carefully to ensure complete resection.

Specimen Retrieval

Resected specimens were retrieved using a basket or net for histopathological examination. If piecemeal resection was necessary, all fragments were collected to ensure complete tissue assessment.

Post-procedure Care

Haemostasis was ensured using adjunctive techniques such as the application of clips or coagulation using haemostatic forceps where necessary. Patients were monitored for immediate complications, and a follow-up endoscopy was scheduled based on histopathology results and procedural findings.

Ethical considerations

All patient data were anonymised for the purpose of this study. No identifying information was retained by the authors or included in this article. Data collection adhered to the principles of the Declaration of Helsinki. Ethical approval was granted by the local audit committee of Maidstone and Tunbridge Wells NHS Trust (approval number: 2737).

All patient data were anonymised for the purpose of this study. No identifying information was retained by the authors or included in this article. Data were analysed using basic descriptive statistics. Ethical approval was granted by the local audit committee.

Statistical analysis

Data were coded and entered using the IBM SPSS Statistics software, version 26 (IBM Corp., Armonk, NY), tested for normality using the Shapiro-Wilk test, and summarised using numbers and percentages for qualitative variables and means and standard deviations for quantitative normally distributed variables. Comparisons between groups were performed using the chi-square test or Fisher’s exact test where appropriate for qualitative variables and independent samples T-test for independent comparisons of quantitative variables. Correlation was used to test linear relationships between variables. A p-value of less than or equal to 0.05 was considered statistically significant. 

## Results

Participant demographics

A total of 350 participants were included in this study. The demographic characteristics of the participants are summarised in Table 1. The cohort comprised 224 males (64%) and 126 females (36%), with a mean age of 70 ± 12 years.

**Table 1:** Socio-demographic characteristics of participants The data in this table are presented as count (N) and percentage (%) unless otherwise stated. Significance was determined at p < 0.05.

Characteristic	Value
Males	224 (64%)
Females	126 (36%)
Mean age	70 ± 12
Total	350

Incidence of complications

Complications were observed in eight participants (2.3%), while 342 participants (97.7%) did not experience any complications. The types and frequencies of complications are detailed in Table 2. The most common complication was bleeding, occurring in three participants (37.5% of those with complications). Other complications included perforation (one participant, 12.5%), vasovagal response (one participant, 12.5%), spasm during the procedure (one participant, 12.5%), withdrawal of consent (one participant, 12.5%), and extreme distress (one participant, 12.5%).

**Table 2:** Types and frequencies of complications The data in this table are presented as count (N) and percentage (%) unless otherwise stated. Significance was determined at p < 0.05.

Complication	Frequency	Percentage (%)
Bleeding	3	37.5
Perforation	1	12.5
Vasovagal response	1	12.5
Spasm during procedure	1	12.5
Withdrawal of consent	1	12.5
Extreme distress	1	12.5

Previous polyp removal attempts

Of the total number of participants, 59 (16.9%) had undergone previous polyp removal attempts, while 291 (83.1%) had no prior attempts.

Polyp characteristics

The mean polyp size was 34.15 ± 22.3 mm. A detailed analysis of polyp size and its relation to complications is provided in Table 3. Participants who experienced complications had a mean polyp size of 49.38 ± 7.3 mm, whereas those without complications had a mean polyp size of 44.85 ± 16.5 mm (p = 0.440).

**Table 3:** Polyp size and complications The data in this table are presented as count (N) and percentage (%) unless otherwise stated. Significance was determined at p < 0.05.

Polyp size	With complications (Mean + SD)	Without complications (Mean + SD)	P-value
in millimeters (mm)	49.98+ 7.3	44.85+ 16.5	0.440

Polyp size categories

Participants were categorised based on polyp size into two groups: <40 mm and ≥40 mm. All complications occurred in participants with polyps ≥40 mm. However, this association was not statistically significant (p = 0.352), as shown in Table 4.

**Table 4:** Polyp size categories and complications The data in this table are presented as count (N) and percentage (%) unless otherwise stated. Significance was determined at p < 0.05. mm: millimeters

Polyp size category	Complications (%)	No complications (%)	p-value
<40 mm	0 (0%)	181 (53.2%)	0.352
≥40 mm	8 (2.3%)	161 (46.8%)	

Polyp multiplicity

Polyp multiplicity was also analysed to assess its association with complications. Among participants with complications, five (62.5%) had single polyps, and three (37.5%) had multiple polyps. In comparison, among those without complications, 146 (42.6%) had single polyps, and 196 (57.4%) had multiple polyps. The difference in complication rates between single and multiple polyps was not statistically significant (p = 0.287) (Table 5).

**Table 5:** Polyp multiplicity and complications The data in this table are presented as count (N) and percentage (%) unless otherwise stated. Significance was determined at p < 0.05.

Polyp multiplicity	With complications	Without complications	p-value
Single	5 (62.5%)	146 (42.6%)	0.287
Multiple	3 (37.5%)	196 (57.4%)	

Correlation analysis

Correlation analysis between polyp size, number of polyps, and complications is summarised in Table 6. The correlation between polyp size and complications was weak (r = 0.095, p = 0.088), and the correlation between the number of polyps and complications was negative and not significant (r = -0.050, p = 0.361).

**Table 6:** Correlation analysis The data in this table are presented as count (N) and percentage (%) unless otherwise stated. Significance was determined at p < 0.05.

Variable	Correlation coefficient (r)	P-value
Polyp size	0.095	0.088
Number of polyps	-0.05	0.361

## Discussion

This retrospective cohort study provides valuable insights into the outcomes of endoscopic excision of large colonic polyps, contributing to the growing body of evidence regarding the safety and efficacy of advanced endoscopic techniques.

Complication rates and safety

The overall complication rate in our study was 2.3%, with bleeding being the most common complication (37.5%). This finding is consistent with Swan et al. (2009) [11], who reported a low complication rate in their prospective study on large refractory colonic polyps managed through a tertiary referral colonic mucosal resection and polypectomy service (TRCPS). Mandic et al. (2022) [11] also noted that complications, particularly bleeding, are a significant consideration in the endoscopic resection of large polyps. Both studies support the conclusion that endoscopic techniques when performed by skilled endoscopists, are safe and effective for the removal of large colonic polyps [11, 12].

Polyp size and complications

In our study, participants with complications had a mean polyp size of 49.38 mm, compared with 44.85 mm in those without complications. Although the difference was not statistically significant (p = 0.440), this trend is noteworthy. Mandic et al. (2022) identified polyp size over 40 mm as a significant predictor of carcinoma presence and recurrence, emphasising the challenges associated with larger polyps [12]. Similarly, Swan et al. (2009) found that larger and more complex polyps were successfully managed with advanced endoscopic techniques, underscoring the importance of expertise in handling such cases to minimise complications [11].

Polyp morphology and recurrence

Polyp morphology is a critical factor in predicting recurrence. In our study, polyp multiplicity did not significantly impact complication rates (p = 0.287). However, Mandic et al. reported that specific morphological types, such as laterally spreading tumour nongranular and type IIB polyps, were significant predictors of recurrence [12]. This suggests that while the number of polyps may not be as critical, their morphological characteristics play a significant role in determining outcomes [13].

Comparison with surgical management

Our findings support the use of endoscopic techniques over surgical management for large colonic polyps. Swan et al. demonstrated that 90% of patients with large or complex polyps avoided surgery when managed through TRCPS, resulting in significant cost savings and reduced morbidity. Similarly, Mandic et al. showed that endoscopic resection is highly effective in preventing the need for surgical intervention, thereby reducing healthcare costs and improving patient outcomes [12]. Lee et al. (2012) compared EMR and ESD and found both techniques to be effective in managing large colorectal polyps, with ESD providing higher en bloc resection rates and lower recurrence rates [13].

Advanced endoscopic techniques

Advancements in endoscopic techniques, such as EMR and ESD, have revolutionised the management of large colonic polyps. Lee et al. (2012) reported that ESD offers a higher rate of complete resection for large and complex polyps compared with EMR, although it is technically more demanding and associated with a higher risk of complications [13]. Tanaka et al. (2015) also highlighted that ESD, despite its technical challenges, provides excellent outcomes for large colorectal polyps when performed by experienced endoscopists [14]. The implementation of quality metrics in endoscopic procedures has been instrumental in standardising care and optimising outcomes [15].

Clinical implications and recommendations

The low complication rates and high success rates observed in our study, as well as in the studies by Swan et al., Mandic et al., and Lee et al., emphasise the importance of skilled endoscopists in managing large colonic polyps [11-13]. Our findings suggest that while larger polyps and specific morphological types may pose higher risks, these can be effectively managed through advanced endoscopic techniques. This supports the need for continued development and training in EMR and ESD, ensuring that endoscopists are sufficiently equipped to handle complex cases.

Limitations

The retrospective nature of our study limits the ability to establish causal relationships. Additionally, the study was conducted within a single NHS trust, which may restrict the generalisability of the findings. Future studies should consider larger, multi-centre cohorts with prospective designs to validate these findings and explore the impact of various polyp characteristics on long-term outcomes.

## Conclusions

Our study demonstrates the efficacy and safety of endoscopic excision of large colonic polyps within a single NHS trust. The findings align with existing literature, supporting the use of advanced endoscopic techniques for managing large and complex polyps. While specific polyp characteristics may increase the risk of complications, these can be effectively managed by experienced endoscopists. Further research is needed to optimise management strategies and validate these findings across different settings.
